# Exosomes Derived From miR-133b-Modified Mesenchymal Stem Cells Promote Recovery After Spinal Cord Injury

**DOI:** 10.3389/fnins.2018.00845

**Published:** 2018-11-22

**Authors:** Dong Li, Peng Zhang, Xiyang Yao, Haiying Li, Haitao Shen, Xiang Li, Jiang Wu, Xiaocheng Lu

**Affiliations:** ^1^Department of Neurosurgery, Lianyungang Hospital of Traditional Chinese Medicine, Lianyungang, China; ^2^Department of Neurosurgery, Brain and Nerve Research Laboratory, The First Affiliated Hospital of Soochow University, Suzhou, China

**Keywords:** exosome, miR-133b, spinal cord injury, axon regeneration, MSCs

## Abstract

Dysregulation of microRNAs (miRNAs) has been found in injured spinal cords after spinal cord injury (SCI). Previous studies have shown that miR-133b plays an important role in the differentiation of neurons and the outgrowth of neurites. Recently, exosomes have been used as novel biological vehicles to transfer miRNAs locally or systemically, but little is known about the effect of the delivery of exosome-mediated miRNAs on the treatment of SCI. In the present study, we observed that mesenchymal stem cells, the most common cell types known to produce exosomes, could package miR-133b into secreted exosomes. After SCI, tail vein injection of miR-133b exosomes into rats significantly improved the recovery of hindlimb function when compared to control groups. Additionally, treatment with miR-133b exosomes reduced the volume of the lesion, preserved neuronal cells, and promoted the regeneration of axons after SCI. We next observed that the expression of RhoA, a direct target of miR-133b, was decreased in the miR-133b exosome group. Moreover, we showed that miR-133b exosomes activated ERK1/2, STAT3, and CREB, which are signaling pathway proteins involved in the survival of neurons and the regeneration of axons. In summary, these findings demonstrated that systemically injecting miR-133b exosomes preserved neurons, promoted the regeneration of axons, and improved the recovery of hindlimb locomotor function following SCI, suggesting that the transfer of exosome-mediated miRNAs represents a novel therapeutic approach for the treatment of SCI.

## Introduction

Traumatic spinal cord injury (SCI) often results in irreversible neurological deficits, with an annual incidence of 15–40 cases per million throughout the world (Sekhon and Fehlings, 2001). Increasing evidence has shown that the spinal cord suffers from primary mechanical injury followed by secondary injury, including inflammation, ischemia, lipid peroxidation, and apoptosis, although the exact pathophysiological mechanisms are still unknown (Popovich, 2014; Stenudd et al., 2015). To date, major progress has been made on neuroprotection and regeneration in preclinical studies;however, finding effective treatments for SCI remains a challenge for basic science and clinical investigators (Dietz and Fouad, 2014).

MicroRNAs (miRNAs) are endogenous ∼22 nucleotide non-coding RNAs that can regulate the expression of protein-coding genes by binding to complementary sites in the 3′-untranslated regions (UTRs) of their target mRNAs (Bartel and Chen, 2004). Recent studies have shown that miRNAs play important roles in synaptic activity, regeneration, and neurogenesis in the central nervous system (CNS). Moreover, several miRNAs have been reported as potentially novel targets for the treatment of SCI, including miR-486, miR-21, and miR-126 (Jee et al., 2012; Hu et al., 2013, 2015). Recently, Yu et al. demonstrated that miR-133b is essential for functional recovery after SCI in zebrafish (Yu et al., 2011). In addition, our previous study indicated that miR-133b promotes the outgrowth of neurites by targeting Ras homolog gene family member A (RhoA) *in vitro* (Lu et al., 2015).

Exosomes are small-membrane vesicles (30–100 nm) derived from the luminal membranes of multivesicular bodies and are secreted from several types of cells (Thery et al., 2002). These extracellular vesicles mediate intercellular communication by transferring miRNAs, mRNAs, DNA, and proteins between cells without direct cell-to-cell contact (Umezu et al., 2014; Lou et al., 2015). Growing evidence suggests that, as intercellular communicators, exosomes act not only locally but also systemically (Katakowski et al., 2013; Lou et al., 2015). Moreover, accumulating studies have demonstrated that exosomes can be manufactured in culture by transferring therapeutic miRNAs to exosome-producing cells; among the cell types known to produce exosomes, mesenchymal stem cells (MSCs) are the most common (Roccaro et al., 2013; Phinney et al., 2015; Long et al., 2017). Therefore, we hypothesized that systemic injection of exosomes derived from miR-133b-modified MSCs could transfer miR-133b into the injured spinal cord and improve functional recovery after SCI.

## Materials and Methods

### Animals

Adult male Sprague–Dawley rats weighing 250–300 g were purchased from the Animal Center of the Chinese Academy of Sciences, Shanghai, China. The animal experimental protocols, including care, breeding, and operative procedures, were approved by the Animal Care and Use Committee of Soochow University and complied with the Guide for the Care and Use of Laboratory Animals approved by the National Institutes of Health.

### Preparation of MSC-Derived miR-133b Exosomes

Primary rat MSCs were isolated from male rats weighing 80–100 g. Briefly, the bone marrow of the femurs and tibias was flushed out with PBS followed by centrifugation. The pellet was suspended in Dulbecco’s modified Eagle medium (DMEM; Life Technologies, United States) with 10% heat-inactivated fetal bovine serum (FBS; Life Technologies) and 1% penicillin–streptomycin, and was then incubated under a humidified atmosphere with 5% CO_2_ at 37°C. The medium was replaced every 3 days, and the MSCs were passaged when the cultures reached 90% confluence.

MSCs were transfected with miR-133b mimic and negative control using Lipofectamine 3000 (Invitrogen, United States) in serum-free medium according to the manufacturer’s instructions. At 72 h after transfection, exosomes were obtained from MSC supernatants using the ExoQuick-TC Kit (System Biosciences, United States). Subsequently, exosome pellets were resuspended in PBS at a total protein concentration of 10 μg/μl. Moreover, the exosomes were characterized by western blotting of exosome surface markers, including CD81, CD63, and CD9. The sequence of the miR-133b mimic was 5’-UUUGGUCCCCUUCAACCAGCUA-3′.

### Compression Spinal Cord Injury Model

Male rats were anesthetized by chloral hydrate (400 mg/kg body weight). Following dissection of the paraspinal muscles, a laminectomy from T9–T11 was performed. Subsequently, SCI was inflicted with an aneurysm clip of 35 g closing force for 60 s at the T10 level as previously described (Figley et al., 2014; Soubeyrand et al., 2014). Finally, the incision was closed in layers with silk sutures. The sham-operated rats only received laminectomy. After surgery, all animals received penicillin and an analgesic for 3 days, and the bladders were manually voided thrice daily. At 24 h following trauma, the animals received treatments by tail intravenous injection of miR-133b exosomes (100 μg exosomes in 0.5 mL of PBS), miR-con exosomes (100 μg exosomes in 0.5 mL PBS), or PBS (0.5 ml) as previously described (Zhang et al., 2015).

### Experimental Groups

The rats were randomly divided into four groups, and testing was performed by blinded observers: (1) sham group (the rats were subjected to sham operation), (2) control group (the rats received SCI and were treated with PBS), (3) miR-con group (the rats were subjected to SCI and treated with miR-con exosomes), and (4) miR-133b group (the rats were subjected to SCI and treated with miR-133b exosomes).

### Western Blot Analysis

Total protein was extracted with RIPA lysis buffer (Beyotime Institute of Biotechnology, China). We used the BCA protein assay kit to assess protein concentrations (Beyotime Institute of Biotechnology). For *in vivo* studies, a 10-mm long segment of the spinal cord containing the injury site was harvested at 4 days following SCI (four animals per group). For western blot analysis, 30 μg of protein was separated by 12% SDS–PAGE and transferred to PVDF membranes (Merck Millipore, Germany). Following blocking with 5% non-fat milk, the membranes were incubated with primary antibodies overnight at 4°C: mouse anti-CD63 (Abcam, Cambridge, United Kingdom), rabbit anti-CD81 (Abcam), rabbit anti-CD9 (Abcam), mouse anti- neurofilament (NF) (Abcam), rabbit anti-growth-associated protein 43 (GAP43) (Abcam), rabbit anti-p-signal transducer and activator of transcription 3 (STAT3) (Cell Signaling Technology, United States), mouse anti-STAT3 (Cell Signaling Technology), rabbit anti-p-cAMP response element-binding protein (CREB) (Abcam), rabbit anti-CREB (Abcam), rabbit anti-RhoA (Cell Signaling Technology), or rabbit anti-β-actin (Cell Signaling Technology). Horseradish peroxidase (HRP)-conjugated goat anti-rabbit or mouse was used as secondary antibody (Cell Signaling Technology). Finally, bands were visualized by enhanced chemiluminescence (ECL) Plus (Thermo Scientific, United States), and the relative band densities were determined by Image Lab (Version 2.0.1).

### Real-Time PCR

Total RNAs from MSCs, exosomes, or spinal cords (four animals per group) were extracted by TRIzol reagent (Invitrogen) according to the manufacturer’s instructions. For *in vivo* studies, a 10-mm long spinal cord segment containing the injury epicenter was harvested. Total RNA was reverse-transcribed to cDNA using the PrimeScript RT reagent kit (TaKaRa, Japan). Quantitative real-time PCR was performed using the miScript SYBR Green PCR kit (QIAGEN, Germany). The relative expression of miRNA normalized to U6 was calculated using the 2^−ΔΔCT^ method.

### Behavioral Assessment

The recovery of hindlimb locomotor function was evaluated by using the Basso–Beattie–Bresnahan (BBB) locomotor rating scale of 0 (no motor activity) to 21 (normal locomotion) (Basso et al., 1995). Two independent investigators blinded to the treatment observed the movement and scored the locomotor function preinjury and at days 1, 3, 5, 9, and 14 post-injury as previously described (Hu et al., 2013; Datto et al., 2015).

### Tissue Processing, Hematoxylin–Eosin (HE) Staining, and Immunohistochemistry

Animals were anesthetized terminally with an overdose of inhalation isoflurane at day 4 after injury. The spinal cords were embedded in an optimal cutting temperature compound. The T9–T11 spinal cord segments near the epicenter of the lesion were collected for histological evaluation. HE staining was performed in accordance with the manufacturer’s instructions to quantify the area of lesion cavity using Imagepro-Plus software. The T9–T11 spinal cord segments in transverse sections were dissected in six rats per group, and every eighth section derived from each animal was used to determine the area of cystic cavity in each group as previously described (Chen et al., 2014; Wang et al., 2014).

For immunohistochemistry, briefly, longitudinal sections containing the lesion site were deparaffinized with xylene and hydrated in graded alcohol, followed by boiling in citrate buffer (pH 6.0) twice for 5 min. Subsequently, to inactivate the endogenous peroxidase, the sections were cooled off and incubated in 3% H_2_O_2_ for 15 min at room temperature. The slides were then blocked with 10% FBS for 10 min and incubated with primary antibodies overnight at 4°C, including rabbit anti-GAP43, rabbit anti-NF, rabbit anti-MBP (Abcam), and mouse anti-NeuN (Abcam). Following washing with TBS, the sections were incubated with fluorescence-labeled secondary antibodies (Abcam). Finally, the sections were stained with DAPI and visualized under a confocal laser-scanning microscope (Olympus LSM-GB200, Tokyo, Japan).

### Statistical Analyses

Data were analyzed with SPSS 19.0 (SPSS Inc., Chicago, IL, United States). All data represent at least three independent experiments and are expressed as means ± standard deviations (SD). One-way analysis of variance (ANOVA) with Tukey’s *post hoc* test was used to compare the levels of different groups. BBB scores were analyzed by repeated measures ANOVA followed by Bonferroni *post hoc* corrections. Statistical significance was set at *p* < 0.05.

## Results

### Reduced Expression of miR-133b in Injured Spinal Cords

**FIGURE 1:**
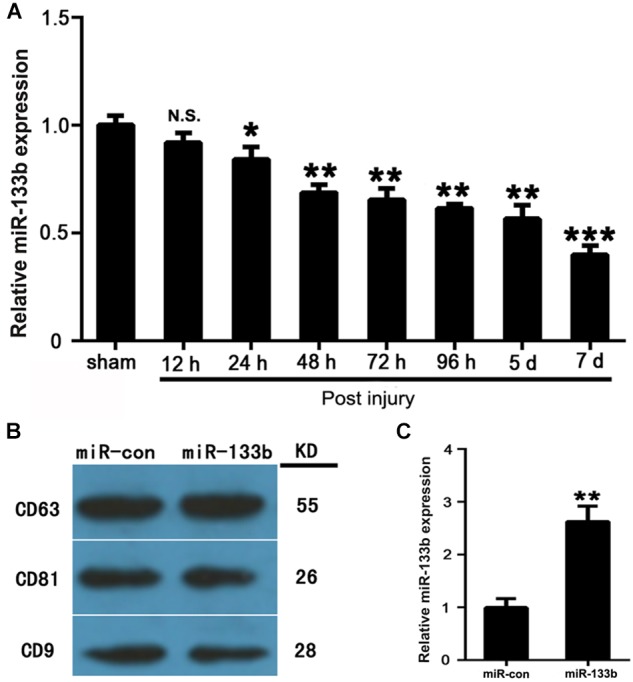
MSCs package miR-133b into secreted exosomes. **(A)** Expression levels of miR-133b in injured spinal cords were measured by qRT-PCR at 12 h, 24 h, and at 2, 3, 4, 5, and 7 days after acute SCI. **(B)** Expressions of CD63, CD81, and CD9 in exosomes derived from miR-133b- or miR-con-transfected MSCs were evaluated by western blot. **(C)** Expression of miR-133b in exosomes derived from miR-133b- or miR-con-transfected MSCs was assessed by qRT-PCR. Values are presented as means ± SD. NS: *p* > 0.05, ^∗^*p* < 0.05, ^∗∗^*p* < 0.01, ^∗∗∗^*p* < 0.001, *n* = 4 per group.

The expression levels of miR-133b in injured spinal cords were measured by qRT-PCR at 12 h, 24 h, 2, 3, 4, 5, and 7 days after acute SCI. The results revealed that the expression of miR-133b was significantly downregulated at 24 h or later following SCI (Figure 1A).

### MSCs Packaged miR-133b Into Secreted Exosomes

MSCs were cultured as described above and characterized as being positive for CD73, CD90, and CD105, but negative for CD34 and CD45 (data not shown). MSCs were then transfected with miR-133b mimic and negative control (the transfection efficiency was approximately 90%). Exosomes were isolated from MSC supernatants at 72 h after transfection. To characterize the exosomes, we carried out western blot analysis, which showed that exosomes expressed common exosomal marker proteins, including CD9, CD63, and CD81 (Figure 1B), as previously described (Roccaro et al., 2013). As shown in Figure 1C, qRT-PCR revealed that the expression levels of miR-133b were approximately 2.5-fold higher in exosomes derived from miR-133b-transfected MSCs than in those from MSCs transfected with miR-con. These results demonstrated that MSCs efficiently packaged miR-133b into secreted exosomes.

### miR-133b Exosomes Improved Functional Recovery, Reduced the Lesion Volume, and Preserved Neurons After SCI

**FIGURE 2:**
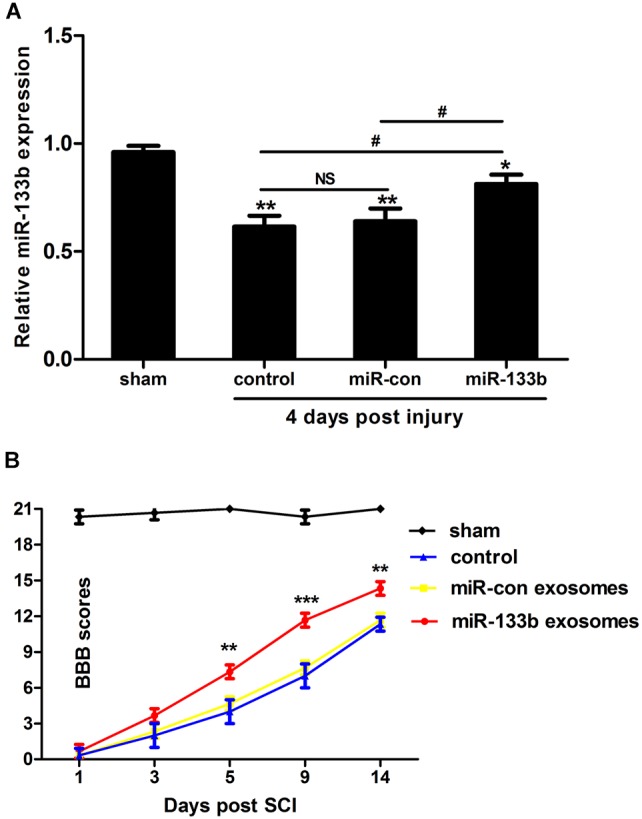
Injection of miR-133b exosomes improved recovery after SCI. **(A)** Relative expression of miR-133b at the lesion site was evaluated by qRT-PCR following various treatments after SCI. Tail vein injection of miR-133b exosomes significantly increased miR-133b expression in the injured spinal cord when compared with control groups. **(B)** Hindlimb functional recovery was monitored at 1, 3, 5, 9, and 14 days after SCI using BBB scoring. Values are means ± SD. NS: *p* > 0.05, ^∗^*p* < 0.05, ^∗∗^*p* < 0.01, ^∗∗∗^*p* < 0.001, ^#^*p* < 0.05, *n* = 4 per group for miR-133b expression and *n* = 6 per group for hindlimb functional recovery.

We first explored using qRT-PCR whether the tail vein injection of exosomes would change the miR-133b expression in spinal cords. As shown in Figure 2A, compared with the control groups, the expression of miR-133b was significantly increased in the miR-133b exosome-injected group at day 4 following SCI.

To investigate whether miR-133b exosomes had a beneficial effect on the recovery of hindlimb locomotor function after acute SCI, the BBB locomotor grading scale was used at different time points after SCI. Immediately after SCI, the BBB score was approximately 0–1, indicating that the SCI model was successful. Spontaneous functional recovery was observed in all groups after SCI, as described previously (Pinzon et al., 2008). After 5 days, improved recovery with significant differences between miR-133b and miR-con exosome-injected rats was observed (day 5, *p* < 0.01; day 9, *p* < 0.001; and day 14, *p* < 0.01), indicating that the tail vein injection of miR-133b exosomes improved the recovery of hindlimb locomotor function after SCI (Figure 2B).

**FIGURE 3:**
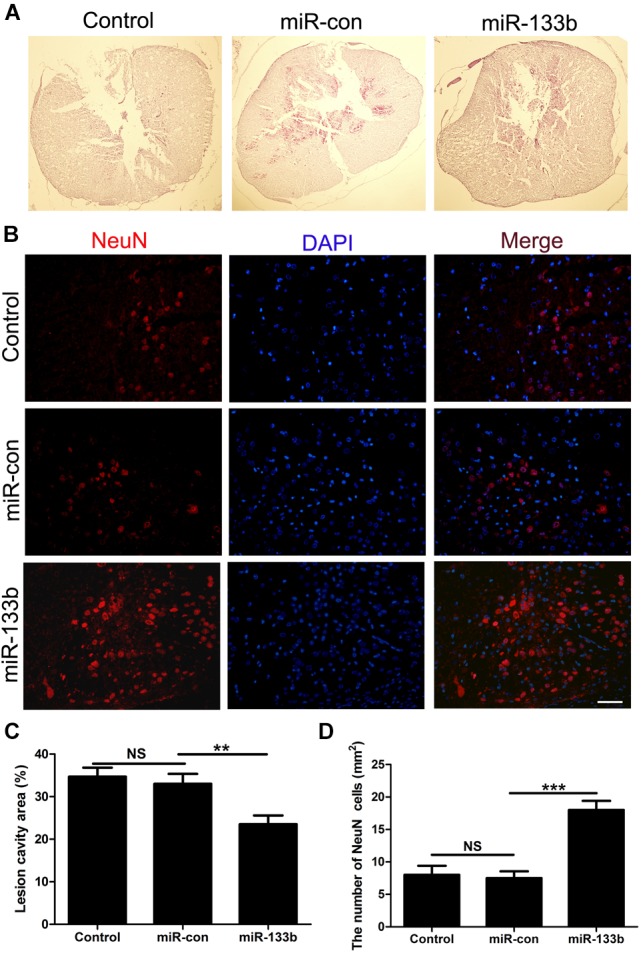
Injection of miR-133b exosomes reduced the lesion volume and preserved NeuN+ neurons after SCI. **(A,C)** HE staining results for sections of injured spinal cord. **(B,D)** The effect of miR-133b exosomes on NeuN+ neurons was evaluated by immunofluorescence staining. Values are means ± SD. NS: *p* > 0.05, ^∗∗∗^*p* < 0.01, ^∗∗∗^*p* < 0.00, Scale bar = 60 μm, *n* = 6 per group.

We next evaluated the effect of miR-133b exosomes on the volume of the lesion and the preservation of NeuN+ neurons after SCI by immunohistochemistry. HE staining showed that miR-133b exosomes significantly decreased the area of lesion cavity when compared to miR-con or the control group (Figures 3A,C). At day 4 after SCI, neurons in the injured spinal cord were stained with antibodies directed against NeuN, a specific marker of mature neurons. As shown in Figures 3B,D, the results showed significantly increased mature neuron numbers in injured rats receiving miR-133b exosomes compared to rats receiving miR-con exosomes (*p* < 0.01).

### miR-133b Exosomes Promoted Axonal Outgrowth After SCI

**FIGURE 4:**
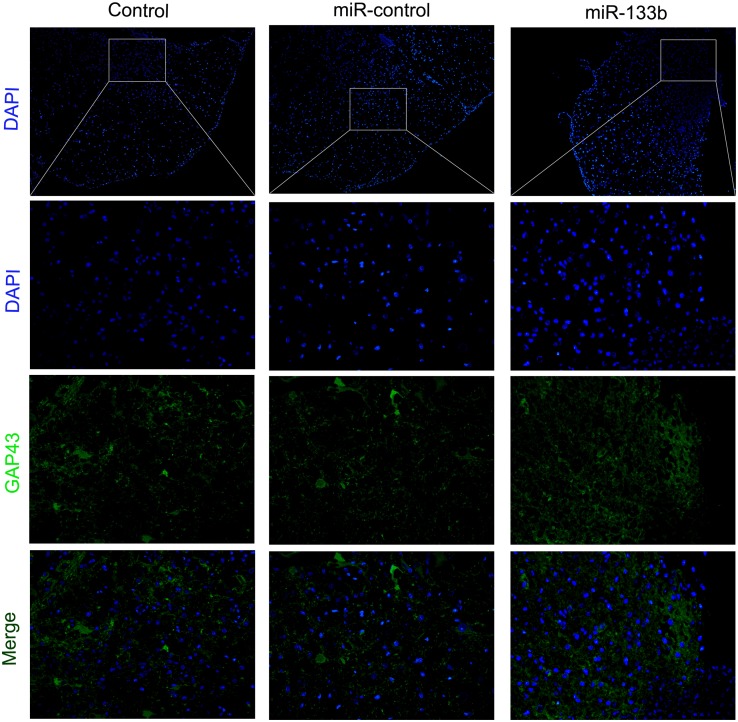
Effect of miR-133b exosomes on GAP43 expression was measured by immunofluorescence staining. Scale bar = 60 μm, *n* = 6 per group.

**FIGURE 5:**
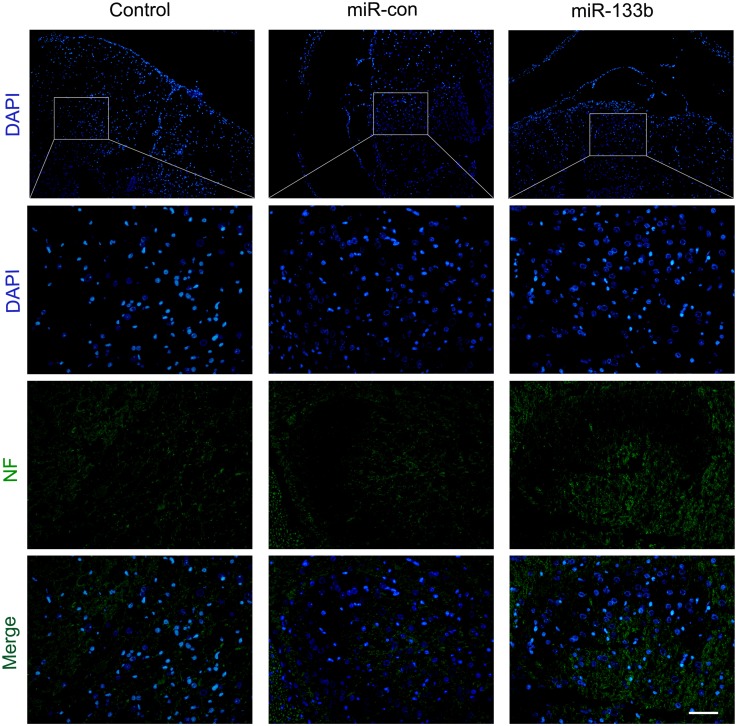
Effect of miR-133b exosomes on NF expression was measured by immunofluorescence staining. Scale bar = 60 μm, *n* = 6 per group.

**FIGURE 6:**
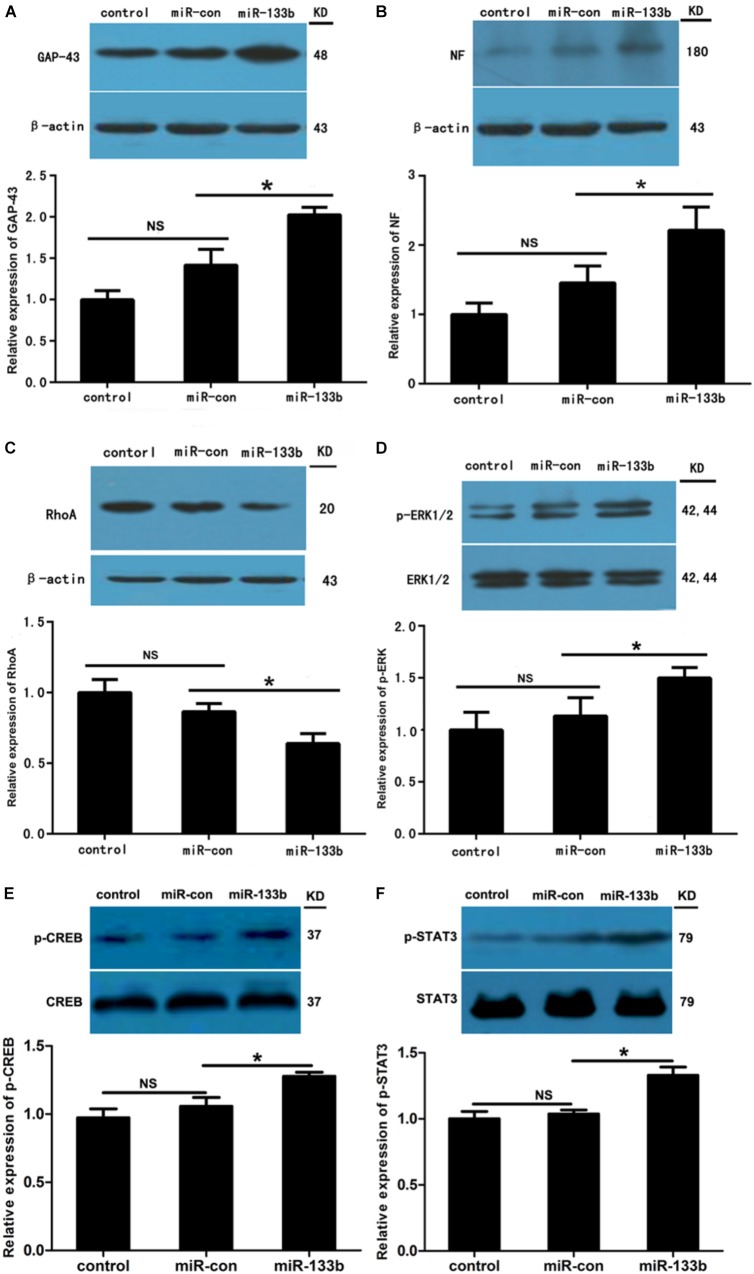
Effect of miR-133b exosomes on expression levels of GAP43, NF, RhoA, pERK1/2, p-CREB, and p-STAT3. Western blotting showed that miR-133b exosomes increased expressions of GAP43 **(A)** and NF **(B)**, reduced the expression of RhoA **(C)**, and promoted phosphorylation of ERK1/2 **(D)**, CREB **(E)**, and STAT3 **(F)** in injured spinal cords after SCI. β-Actin was used as a control for GAP43, NF, and RhoA, and ERK1/2, CREB, and STAT3 were used as controls for pERK1/2, p-CREB, and p-STAT3, respectively. Values are presented as means ± SD. NS: *p* > 0.05, ^∗^*p* < 0.05, *n* = 4 per group.

To investigate the potential effect of miR-133b exosomes on the outgrowth of axons, an immunohistochemistry study of GAP43 and NF was carried out (Van der Zee et al., 1989). The results showed that the expression of GAP43 was increased in the miR-133b exosomes group compared to the miR-con or control group (Figure 4). Moreover, we observed that treatment with miR-133b exosomes enhanced NF expression at day 4 after SCI (Figure 5). Finally, western blot results confirmed significant increases in the protein levels of GAP43 and NF in the miR-133b exosomes group compared with those in the miR-con group (Figures 6A,B).

### Effect of miR-133b Exosomes on Neuroprotection-Related Pathways and Phosphorylation of CREB and STAT3

Our previous study indicated that RhoA is a direct target of miR-133b (Lu et al., 2015). Moreover, RhoA has been shown to be involved in the death of neuronal cells in the spinal cord (Wu et al., 2016). To assess the effect of miR-133b exosomes on the expression of RhoA after SCI, we detected the levels of RhoA expression by using western blot. The results demonstrated that the expression of RhoA was significantly decreased in the injured spinal cords of rats receiving miR-133b exosome injections compared to those in miR-con exosome-treated animals (*p* < 0.05) (Figure 6C). These results are consistent with those of other *in vitro* and *in vivo* studies (Niu et al., 2016; Theis et al., 2016). Moreover, the phosphorylation of ERK1/2, another cell survival-related pathway protein, was significantly increased in the injured rats treated with miR-133b exosomes after SCI (Figure 6D).

The transcription factors CREB and STAT3 have been shown to be involved in the outgrowth of neurites (Gao et al., 2004; Qiu et al., 2005). In this study, we observed that injecting miR-133b exosomes increased the protein phosphorylation levels of STAT3 and CREB in injured spinal cords after SCI (*p* < 0.05 for p-STAT and *p* < 0.05 for p-CREB; Figures 6E,F).

## Discussion

SCI has a significant impact on both the patient and society (McDonald and Sadowsky, 2002). Following SCI, functional recovery is often poor, and to date there have been no effective therapies that have been translated to the clinic (Ramer et al., 2014). Increasing evidence has demonstrated that miRNAs are involved in the pathogenesis of SCI. For instance, miR-21 has been shown to decrease the death of neuronal cells and promote functional recovery after SCI (Hu et al., 2013). Recent studies have indicated that miR-133b plays important roles in neuronal differentiation, neurite outgrowth, and neuronal apoptosis in the CNS (Heyer et al., 2012; Lu et al., 2015; Niu et al., 2016; Xia et al., 2016). Moreover, further studies have shown that overexpression of miR-133b improves functional recovery after stroke in rats (Xin et al., 2013b, 2017). Exosomes, a novel intercellular communicator, have been used as biological vehicles for local or systemic delivery of miRNAs in the treatment of various diseases, such as stroke and Parkinson’s disease (Xin et al., 2013a; Haney et al., 2015). In the current study, we investigated the effect of the transfer of exosome-mediated miR-133b in the treatment of SCI. After SCI, we observed significant differences in the recovery of functions between the miR-133b exosome group and the control group after SCI. Previous studies indicated that various treatments could improve the functional recovery from 3 days after SCI (Paterniti et al., 2014; Liu et al., 2015). In this study, we observed that, after 5 days, BBB scores were significantly higher in animals systemically injected with miR-133b exosomes than in the miR-con-injected rats. These findings are consistent with a recent study that demonstrated that lentiviral delivery of miR-133b improves functional recovery after SCI in mice (Theis et al., 2016).

Several mRNAs have been reported as targets of miR-133b, such as RhoA and MST2 (Care et al., 2007; Qin et al., 2012). In the present study, we found a reduction of RhoA protein levels in the injured spinal cords of rats receiving miR-133b exosomes. This finding is consistent with previous *in vitro* studies, indicating that RhoA is a direct target of miR-133b (Lu et al., 2015; Niu et al., 2016). RhoA, a member of the Rho family, has been shown to be upregulated after SCI in rats and acts on its direct downstream effector Rho-associated kinase (ROCK) (Schwab et al., 2005). Since a recent study demonstrated that the RhoA/ROCK signaling pathway plays a critical role in the death of spinal cord neurons after acute SCI (Wu et al., 2016), we next tested whether the survival of neurons was enhanced *in vivo* after miR-133b exosome injection. After SCI, neuronal death occurs at the lesion site within 24 h and is attributed to the primary mechanical force and later secondary factors such as inflammation, oxidation, and apoptosis (Liu et al., 1997; Esposito et al., 2012; Sonmez et al., 2013). In this study, at day 4 following SCI, we observed that the number of mature neurons was significantly increased in the miR-133b exosomes group. These findings are consistent with a recent study that demonstrated that the inhibition of RhoA significantly reduces the death of neuronal cells after SCI (Wu et al., 2016). In addition, we also observed that the injection of miR-133b exosomes enhanced ERK1/2 phosphorylation at the lesion site after SCI. Our previous *in vitro* study indicated that knockdown of RhoA protein by siRNA enhances the phosphorylation of ERK1/2 in PC12 cells, consistent with previous reports (Li et al., 2013; Gordon et al., 2014; Lu et al., 2015). Thus, miR-133b may promote the phosphorylation of ERK1/2 by targeting RhoA. The ERK pathway has also been shown to be crucial for the survival of neuronal cells after SCI. Previous studies have indicated that the activation of ERK1/2 protects neurons from apoptosis and improves the recovery of functions after SCI (Yune et al., 2008; Lee et al., 2010). These results suggest that the neuroprotective effect of miR-133b exosomes might involve the inhibition of RhoA and the activation of ERK1/2.

It has been established that the lack of regeneration of axons in the injured CNS is largely due to the presence of inhibitory molecules, including oligodendrocyte myelin glycoprotein, myelin-Nogo, and myelin-associated glycoprotein. To investigate the effect of miR-133b exosomes on the outgrowth of neurites, the spinal cord was analyzed by using immunohistochemical staining for NF. We observed that NF expression was elevated in the miR-133b exosomes group and this agreed with the results of western blotting. Next, we examined the expression of GAP43, a regeneration-associated gene that is upregulated in regenerating neurons (Woolf et al., 1990). A significant increase in the expression of GAP43 was observed in the injured spinal cords of rats injected with miR-133b exosomes at day 4 following SCI, suggesting that miR-133b exosomes promoted the regeneration of axons in injured spinal cords.

Growing evidence has shown that the transcription factor CREB plays important roles in the regeneration of axons (Filbin, 2003). The activation of CREB has been shown to be sufficient to overcome myelin inhibitors and to promote the regeneration of spinal axons *in vivo* (Gao et al., 2004). Here, we found that injecting miR-133b exosomes activated CREB in injured spinal cords. Moreover, the phosphorylation of STAT3, which is involved in the regeneration of axons in the spinal cord, was significantly increased in injured rats treated with miR-133b exosomes compared with those in the miR-con exosomes group (Qiu et al., 2005). These results suggested that miR-133b exosomes enhanced the regeneration of axons after SCI, at least partially, by promoting the phosphorylation of CREB and STAT3.

In conclusion, the results of this study showed that systemically injecting miR-133b exosomes upregulated miR-133b expression at the lesion site and promoted functional recovery after SCI. Moreover, we observed that miR-133b exosomes preserved neuronal cells and enhanced the regeneration of axons, which was attributed at least partially to the activation of ERK1/2, STAT3, and CREB, as well as to the inhibition of RhoA expression. These results suggest that systemic injection of exosomes generated from miRNA-modified MSCs represents a novel therapeutic approach for SCI.

## Author Contributions

XLu and JW designed the study. DL, PZ, XY, HL, HS, and XLi performed the experiments and analyzed the data. XLu wrote the paper. All the authors read and approved the manuscript.

## Conflict of Interest Statement

The authors declare that the research was conducted in the absence of any commercial or financial relationships that could be construed as a potential conflict of interest.
